# Green Synthesis of Zinc Oxide Nanoparticles Using *Dillenia Indica* and *Mikania Micrantha* Leaf Extracts: Applications in Photocatalysis and Antibacterial Activity

**DOI:** 10.1002/open.202400102

**Published:** 2024-10-02

**Authors:** Protap Kumar Pal, Md. Sarifujjaman, Prianka Saha, S. M. Mahbubur Rahman, Md. Emdadul Islam, Bashir Ahmmad, Kaykobad Md. Rezaul Karim, Md. Mahiuddin

**Affiliations:** ^1^ Chemistry Discipline Khulna University Khulna 9208 Bangladesh; ^2^ Biotechnology and Genetic Engineering Discipline Khulna University Khulna 9208 Bangladesh; ^3^ Graduate School of Science and Engineering Yamagata University 4–3-16 Jonan Yonezawa 992–8510 Japan

**Keywords:** Antibacterial agents, Organic dyes, Plant extracts, Photocatalysts, Zinc oxide nanoparticles

## Abstract

Researchers are keenly interested in developing metal‐based nanoparticles using plant sources as they are eco‐friendly, less expensive and simpler. Zinc oxide nanoparticles, symbolized as D‐ZnONPs and M‐ZnONPs were synthesized in this study utilizing the leaves of *D. indica* and *M. micrantha*, respectively, and studied their impact on the growth inhibition of various bacterial strains and on the photocatalysis. By displaying the distinctive surface plasmon resonance (SPR) band at 373 nm in UV‐Vis and bands at 450–480 cm^−1^ corresponding to Zn−O stretching FTIR spectroscopy imparted the formation of ZnONPs which was further supported by X‐ray diffraction analysis by showing the polycrystalline nature and a hexagonal wurtzite structure. The spherical form and average particle size of 30 nm of the produced ZnONPs, as confirmed by electron microscopy, are also confirmed to be crystalline. Under natural sunlight, both ZnONPs demonstrate excellent degradation efficacy about 96–99 % within 100 min towards methylene blue (MB). Furthermore, it is noteworthy that both the synthesized ZnONPs exhibited 55–60 % efficacy with respect to antibiotics in inhibiting the growth of various pathogenic bacterial strains. Overall, ZnONPs can be produced on a large‐scale using plant sources and employed them in environmental remediation and cosmetic industries as prominent components.

## Graphical abstract

Eco‐friendly synthesis of ZnONPs using leaf extract of D. indica and M. micrantha. Both of the ZnONPs are poly polycrystalline having hexagonal wurtzite structure, and consist of spherical and polyhedral shaped particles of 25–30 nm in size for spherical ones. Obtained ZnONPs are excellent photocatalysts for the degradation of methylene blue (MB) dye under solar irradiation and have significant activity against various pathogenic bacteria.

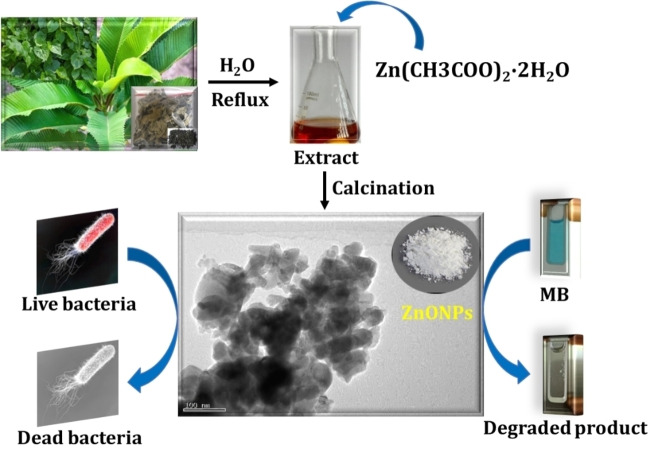

## Introduction

Nanomaterials are more significant than their bulk form because of their unique and sophisticated features and cutting‐edge qualities, which have a functional influence on all scientific domains.[^1^, ^2^, ^3^, ^4^] Nanomaterials have a wide range of applications due to their special, advantageous chemical, physical, and mechanical characteristics.[^1^, ^5^, ^6^, ^7^, ^8^, ^9^, ^10^, ^11^, ^12^] Numerous research teams are currently devoting endless hours to the discovery and development of novel nanomaterials in a variety of methods, investigating their potential new uses.[^13^, ^14^, ^15^, ^16^] Materials containing nanostructured components with diameters less than 100 nm are referred to as nanomaterials.[^17^, ^18^] Metal nanoparticles (NPs) are extensively employed in several nanotechnology domains such as sensors, microelectronics, catalysis, and large surface area because of their small size, robust electrical conductivity, and advantageous chemical and optical properties.[^10^, ^19^, ^20^, ^21^] Zinc oxide nanoparticles (ZnONPs) are one type of nanomaterial that has caught the interest of many researchers due to their easily tunable optical and chemical properties through morphological modification.[^22^, ^23^, ^24^, ^25^] ZnONPs are a member of the broad family of metal oxide nanoparticles and have found utility in a number of cutting edge fields, including biology, electronics, communication, sensors, cosmetics, and the medical field.[^24^, ^26^, ^27^, ^28^] ZnONPs also have a great deal of potential for bioremediation, antibacterial uses, and catalysis.[^6^, ^29^, ^30^, ^31^, ^32^, ^33^, ^34^]

Researchers have recently shown a greater interest in the environmentally friendly synthesis of NPs because of its low cost, ability to be synthesized in ambient conditions, lack of toxicity, compatibility with various environments, and ease of application due to the highly soluble in water, biocompatible, and lack of toxic stabilizers of the final particles.[^35^, ^36^] Extracts from plant‐sources are a very promising components for easy green pathways NPs synthesis. Furthermore, using the redundant components of plant as a source of green synthesis improves the sustainability and appeal of the synthetic process. Many researchers have employed extracts from *Hibiscus subdariffa* leaf,^[37]^ *Garcinia xanthochymus* fruit,^[30]^ *Cassia fistula* and *Melia azedarach* leaf,^[38]^ *Eucalyptus globulus* leaf,^[39]^ *Syzygium Cumini* leaf,^[40]^ orange fruit peel,^[41]^ *Myristica fragrans* fruit,^[42]^ banana peel,^[43]^ *Passiflora foetida* peel,^[44]^ *Lippia adoensis* leaf,^[31]^ *Punica granatum* peel,^[29]^ and so on to synthesize ZnONPs and utilized the obtained ZnONPs in different applications. With no end in sight, the researchers intend to employ various plant sources to produce ZnONPs with more sophisticated qualities that will help them in a range of applications. Our study focuses on the synthesis of ZnONPs using plant sources and their intriguing applications because of this sort of optimism.

*M. micrantha* and *D. indica* are common plants known as Japani lota/American rope plant and chalta/elephant apple and belong to the *Asteraceae* and *Dilleniaceae* families, respectively. These plants are inexpensive and weed plants are found in abundance in South and Southeast Asia, especially in Bangladesh. Research indicates that *M. micrantha* has a number of therapeutic benefits, including antioxidant, antibacterial, analgesic, anti‐inflammatory, and antidiabetic effects.[^45^, ^46^] *M. micrantha* leaves contain a wide range of phytochemicals, such as lactones, steroids, flavonoids, diterpene glucosides, and phenolic compounds such as 3,4‐dihydroxybenzoate, ethyl 2,3‐dihydroxybenzoate, E‐3‐(3,4‐dihydroxyphenyl) acrylic acid, 4‐allyl‐2,6‐dimethoxyphenol glucoside, (+)‐isolariciresinol, icariol A2, 9,10‐dihydroxythymol, 8,9,10‐trihydroxythymol, caffeic acid, p‐coumaric acid, ethyl protocatechuate, procatechuic aldehyde, hydroquinone, etc.^[47]^ Since ancient times, the pulp of the *D. indica* fruit has been applied to the scalp to treat hair loss and dandruff, while the sepal has been used to heal gastrointestinal problems. Sesquiterpene lactones, flavonoids, steroids, diterpene glucosides, and phenolic compounds like 2‐caffeoylisocitric acid, naringenin, kaempferol, 5,7‐dimethoxyapigenin, bryaflavan, mallotus B‐glucose adduct, 11‐dodecenoic acid, nutriacholic acid, etc. are among the many phytochemicals found in *D. indica*.^[48]^

The aforementioned phytochemicals have proven to be effective in the synthesis of several metal‐based stable nanoparticles by acting as reducing and stabilizing agents. For examples, *D. indica* leaf extract has already been used to synthesize silver, selenium, cerium oxide, and gold nanoparticles;[^49^, ^50^, ^51^, ^52^] *M. micrantha* leaf extract was used to synthesize silver and magnetic iron oxide nanoparticles.[^53^, ^54^] For the synthesis of ZnONPs, presumably, the same reduction mechanism would apply. As of right now, there are no reports on the synthesis of ZnONPs employing *D. indica* and *M. micrantha* aqueous leaf extracts.

Addressing water contamination by organic dyes and bacteria requires a multifaceted approach involving advanced technologies, sustainable practices, and innovative materials. The integration of nanoparticles and multifunctional treatment systems holds promise for effective and eco‐friendly remediation of contaminated water. Combining photocatalytic and antibacterial properties in nanoparticles provides a dual‐action approach to tackle both dyes and bacteria simultaneously. ZnONPs are effective photocatalysts that, under UV light, can degrade dyes and exhibit antibacterial properties. Green‐synthesized ZnONPs often exhibit improved photocatalytic and antibacterial activities due to the presence of bioactive compounds from plant extracts.[^29^, ^32^, ^55^, ^56^]

Therefore, the primary goal of this study is to synthesize ZnONPs utilizing a green technique employing the aqueous extract of *D. indica* and *M. microntha* leaves. To varify their sutability in wastewater treatment, the resulting ZnONPs were then employed as an antibacterial agent against different bacterial strains and as photocatalysts to degrade methylene blue (MB), a model organic dye, by harnessing sunlight.

## Results and Discussion

### Formation of ZnONPs

**Figure 1:**
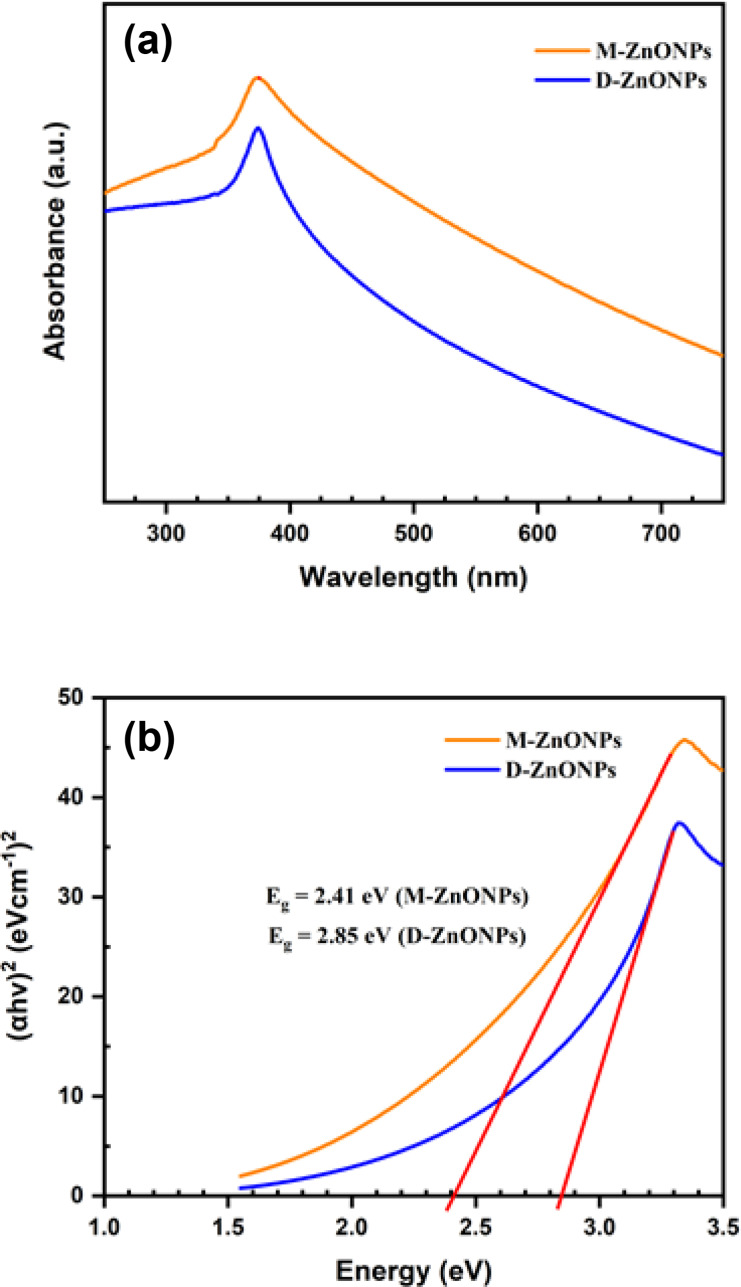
(a) UV‐Vis absorption spectrum of ZnONPs synthesized *using M. micrantha & D. indica* leaf extract and (b) Tauc plot for determining the optical band gap energy of synthesized ZnONPs.

Ultraviolet‐visible (UV‐Vis) spectroscopy has been a key technique in characterizing the metal‐based nanoparticles. It gives the distinctive absorption band of nanoparticles and aids in using the Tauc plot to compute the optical band gap. In order to describe the green ZnONPs that were synthesized utilizing leaf extract from *D. indica* and *M. micrantha*, UV spectroscopy was utilized. The UV‐Vis absorption spectra of D‐ZnONPs and M‐ZnONPs, which were produced with the help of leaf extract from *D. indica* and *M. micrantha*, respectively, are displayed in Figure 1a. Both of the obtained products show the absorption band at 373 nm, which is a distinctive feature of ZnONPs[^30^, ^37^, ^39^] and indicates that *D. indica* and *M. micrantha* leaf extract assisted in the synthesis of ZnONPs. The formation of monodispersed ZnONPs is supported by the building of sharp and intense absorption peaks^[39]^ and absence of any other peaks support the formation of literally pure ZnONPs.

Understanding and tuning the band gap energy of ZnONPs is vital for optimizing their performance in optoelectronic, photocatalysis, solar cells, sensors, and biomedical applications, making it a central focus of research and development in nanotechnology and materials science. Here, by using the Tauc's expression (equation 1), the energy associated with the optical band gap was determined from UV‐Vis absorption spectra:

(1) $$\alpha hv=B{\left(hv-E_{g}\right)}^{m}$$

where α is the absorption coefficient, h is Planck's constant, ν is photon frequency, B is a constant, E_g_ is the optical band gap and m=2 or 1/2 for direct and indirect allowed transitions, respectively.^[57]^ The band gap energy or Tauc optical band gap energy was determined by extrapolating the linear trend to the x‐axis of the (*αhν*)^2^ versus the hv plot (Figure 1b). The D‐ZnONPs and M‐ZnONPs obtained here have a bandgap energy of 2.85 and 2.41 eV, respectively, which is smaller than the value of bulk ZnO (3.45 eV). Due to ZnONPs′ strong UV absorption, these substances could gain excellent acceptability as a photocatalyst, as antimicrobial agents in ointments, and as UV light filter in sunscreen cream.[^58^, ^59^]

**Figure 2:**
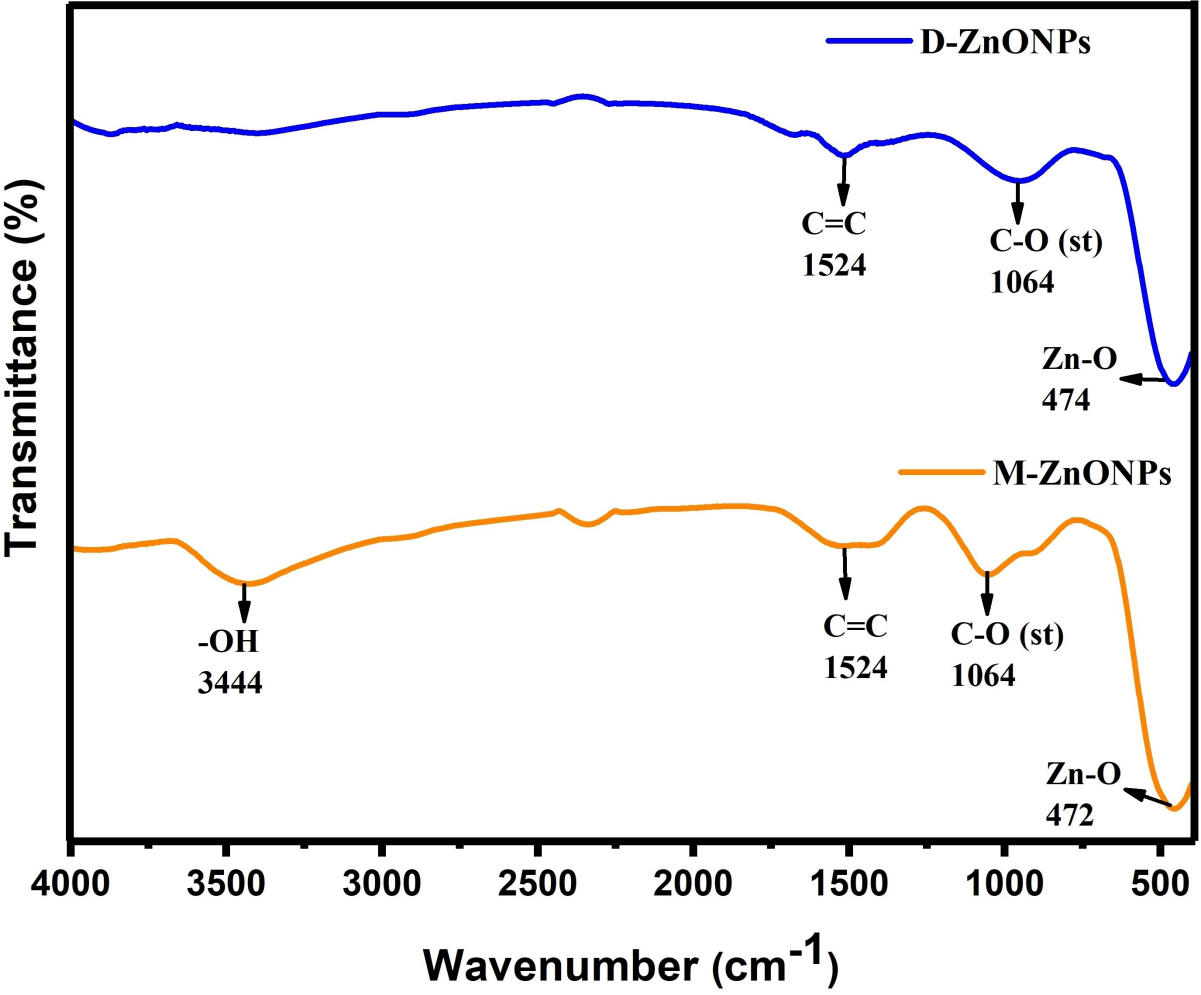
FTIR spectra of ZnONPs synthesized using *M. micrantha & D. indica* leaf extracts.

Fourier transform infrared (FTIR) spectroscopic analysis is a critical technique for characterizing the chemical structure, surface chemistry, and functionalization of ZnONPs, providing essential information for their effective application and further development. In plant‐sources based green synthesis, FTIR spectroscopy have been utilized to examine the chemical composition of plant‐sources that presumably participated in the bio‐reduction of metal ions and stabilize the obtained nanoparticles as well. Furthermore, in the case of metal oxide nanoparticles, it could be useful in anticipating the development of metal‐oxygen bong. Therefore, in order to assume that Zn−O was formed from zinc salt, FTIR spectroscopic study was performed on synthesized products. The Zn−O stretching bonds in the FTIR spectra of D‐ZnONPs and M‐ZnONPs are as shown in Figure 2. These peaks are at 450–480 cm^−1^ and is consistent with early findings in the literature that point to the synthesis of ZnONPs.[^40^, ^41^] However, there are also some peaks in the FTIR spectra about 1000–1064 cm^−1^ and 1510–1525 cm^−1^. These bands show that the plant extract contains C−O−H or C=C groups, indicating the presence of certain phytochemicals on the surface of ZnONPs.

**Figure 3:**
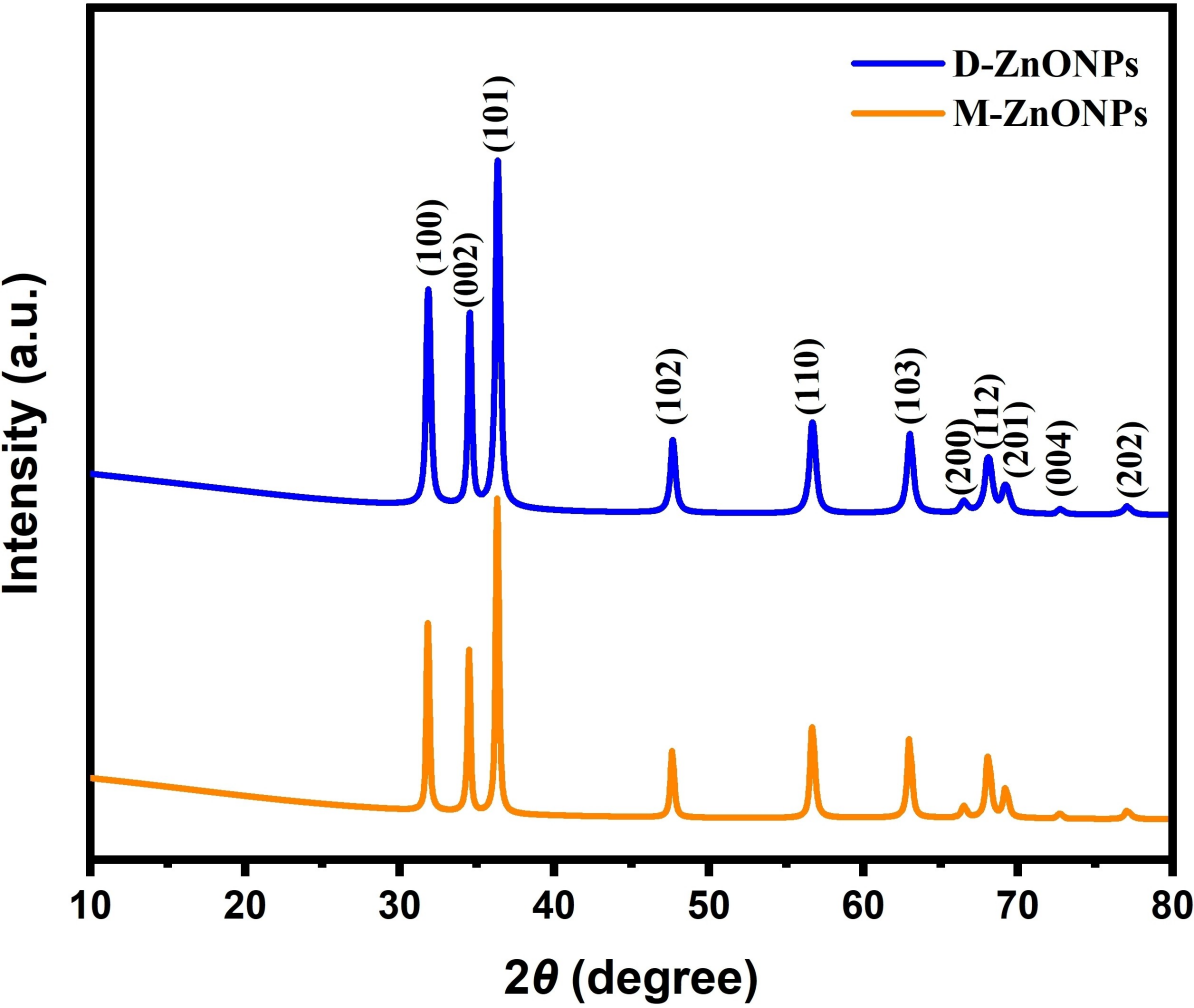
XRD patterns of ZnONPs synthesized using *M. micrantha & D. indica* leaf extracts.

The X‐ray diffraction (XRD) analysis is a key analytical technique that is essentially required to forecast the phase purity, particle size, and crystalline nature of metal‐based nanomaterials. In order to verify the formation, crystallographic nature, and phase purity of the D‐ZnONPs and M‐ZnONPs that were synthesized using *D. indica* and *M. micrantha* leaf extract, respectively, XRD analysis was conducted. Both of these XRD patterns are shown in Figure 3. The major and significant diffraction peaks, which correspond to the (100), (002), (101), (102), (110), (103), (200), (112), (201), (004), and (202) planes, are observed at 31.80°, 34.47°, 36.29°, 47.60°, 56.66°, 62.95°, 66.33°, 67.9°, 69.12°, 72.95°, and 77.85°, respectively. They also match the JCPDS‐36‐1451 well, indicating the formation of crystalline ZnONPs with a hexagonal wurtzite phase. The diffractograms showed no distinctive peak attributed to any contaminant, indicating the synthesis of pure crystalline ZnONPs.[^42^, ^43^]

The average crystallite size of ZnONPs was calculated using the well known Debye–Scherrer formula2, 3

(2) D=kλ/βcosθ

(3) D=0.9λ/βcosθ

Where, λ is the wavelength,

θ is the Bragg's diffraction angle.

β is the full‐width half maximum of the XRD pattern.

K is the Scherrer constant value from 0.9 to 1.

It was revealed that the average crystallite size was obtained 23 nm and 30 nm for D‐ZnONPs and M‐ZnONPs, respectively.

**Figure 4:**
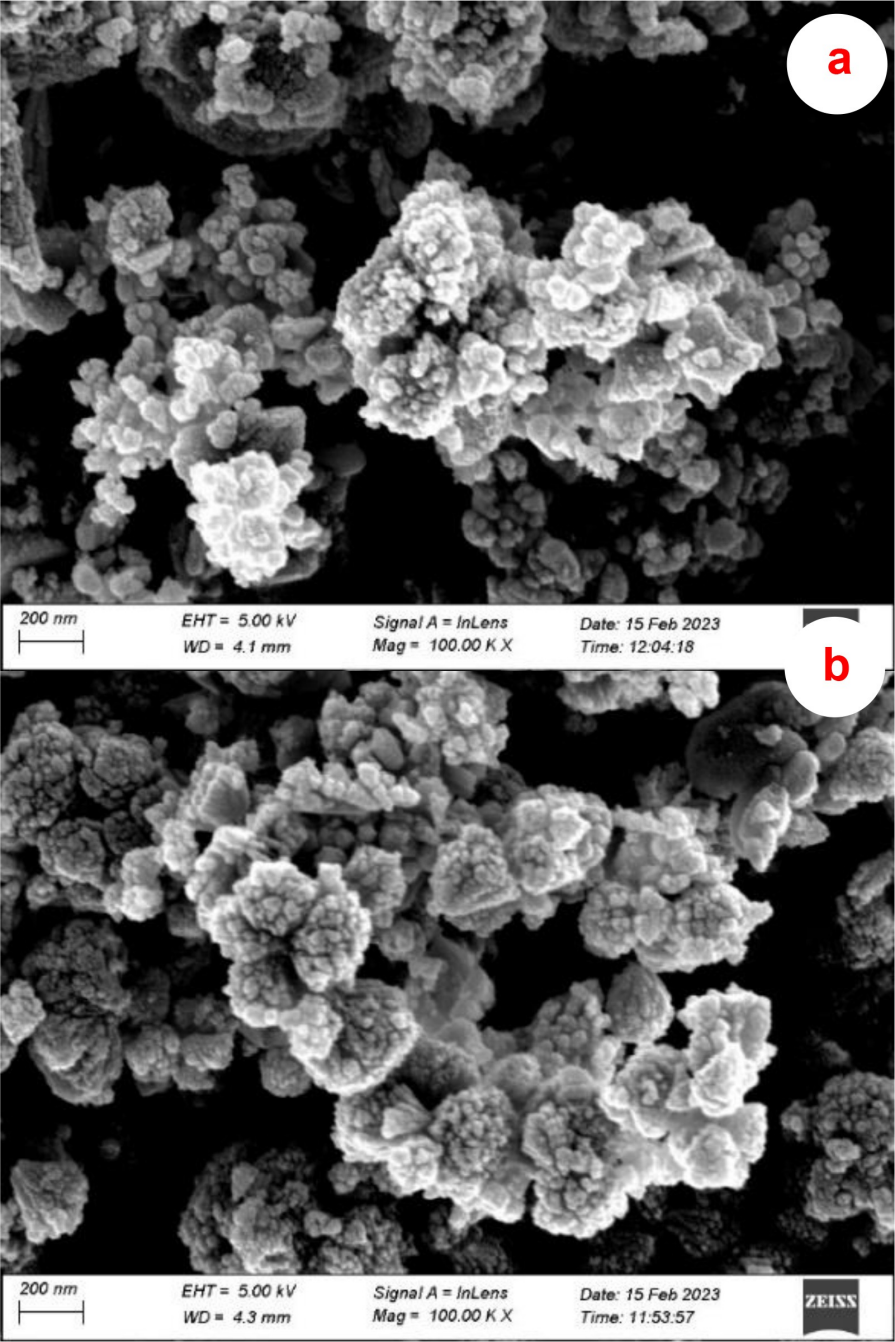
SEM images of ZnONPs synthesized using (a) *D. indica* and (b) *M. micrantha* leaf extract.

The most effective and powerful tool for analyzing the chemical and structural characteristics at the nanoscale is electron microscopy. Thus, the produced D‐ZnONPs and M‐ZnONPs utilizing *D. indica* and *M. micrantha* leaf extract, respectively, were characterized using scanning electron microscopy (SEM) and transmission electron microscopy. Figures 4a and 4b of the SEM images of D‐ZnONPs and M‐ZnONPs, respectively, show the spherical particles with high homogeneity. Because the dry sample was used for analysis, some aggregation was seen. We made an effort to determine the size of the produced ZnONPs, despite the difficulty in doing so from the SEM image. The size of the produced D‐ZnONPs and M‐ZnONPs were found to be within a wide range using ImageJ software, with most particles falling between 20 and 40 nm and a small number largerr than 100 nm, making it impracticle to determine the average particle size.

**Figure 5:**
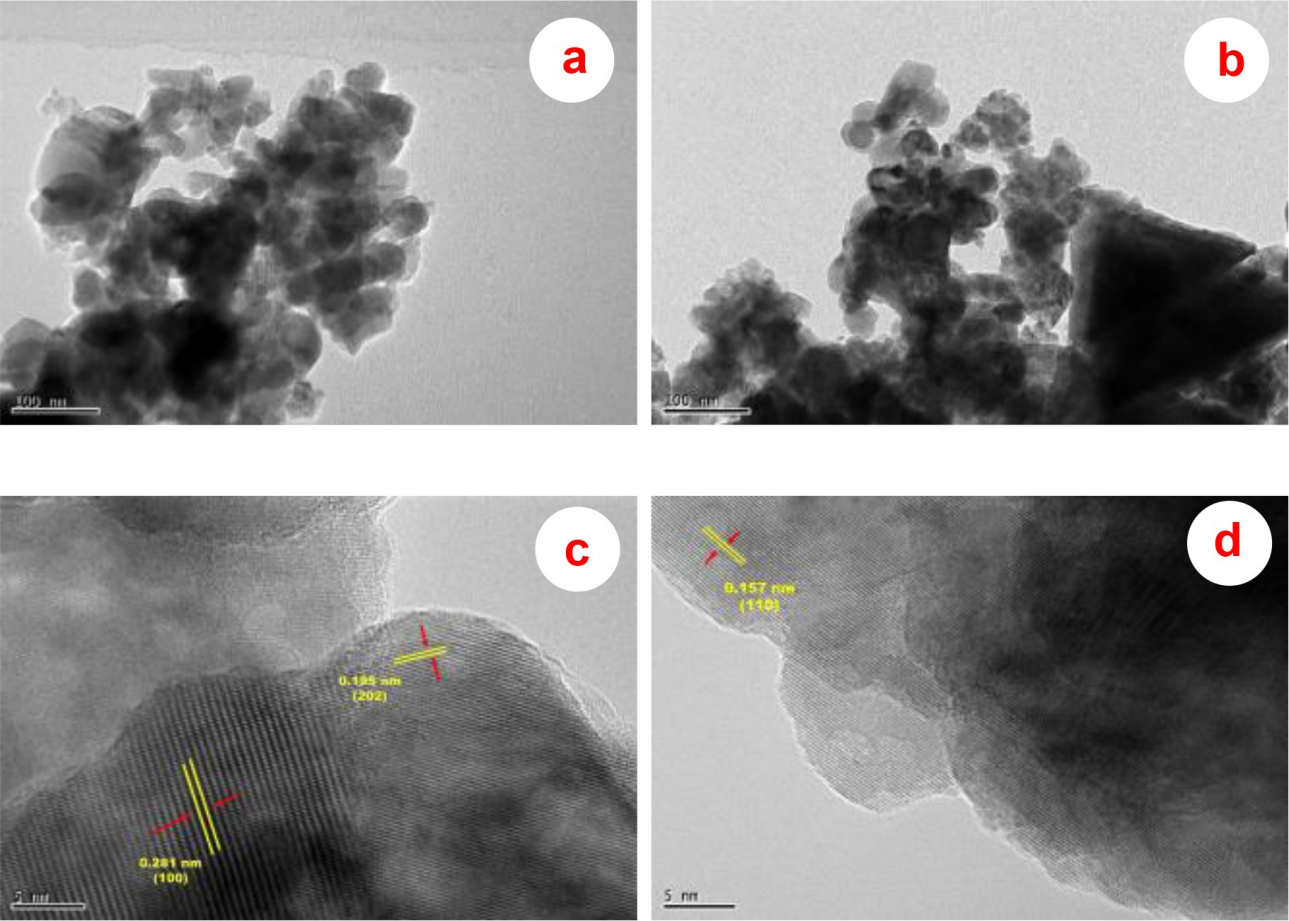
TEM images of (a) D‐ZnONPs and (b) M‐ZnONPs; HRTEM images of (c) D‐ZnONPs and (d) M‐ZnONPs.

TEM examination was also carried out to further examine the shape of the ZnONPs that were produced utilizing the leaf extracts of *D. indica* and *M. micrantha*. The TEM images of the produced D‐ZnONPs and M‐ZnONPs are displayed in Figures 5a and 5b, respectively. The TEM analysis of both ZnONPs reveals the existence of irregularly shaped and spherical particles. The spherical particles of D‐ZnONPs and M‐ZnONPs had an average particle size of 30 and 26 nm, respectively. This result aligns with the outcomes of XRD and SEM analyses. Despite the tiny size of the particles, there is a noticeable aggregation of them. This is most likely because of the high basic condition that was kept throughout the synthesis process. Because there is more dissolved OH^−^ at higher pH levels (>9), ZnO crystals react with too much OH^−^, which causes the ZnO to disintegrate. The crystallites and particles agglomerated and became smaller as a result of this disintegration.^[32]^ Figures 5c and 5d display the HRTEM images of D‐ZnONPs and M‐ZnONPs, respectively. Clear lattice fringes can be seen in the HRTEM images, confirming the great crystallinity of the produced ZnONPs. The lattice fringes of the typical crystallite in the HRTEM image of D‐ZnONPs (Figure 5c) are 0.159 nm and 0.281 nm, respectively. These values are in good agreement with the distances of the (202) and (100) planes of the hexagonal wurtzite ZnO. However, the lattice fringe of the typical crystallite, which measure 0.157 nm, is shown in the HRTEM image of M‐ZnONPs (Figure 5d), and they correspond well with the distance of the (100) plane of hexagonal wurtzite ZnO.

**Figure 6:**
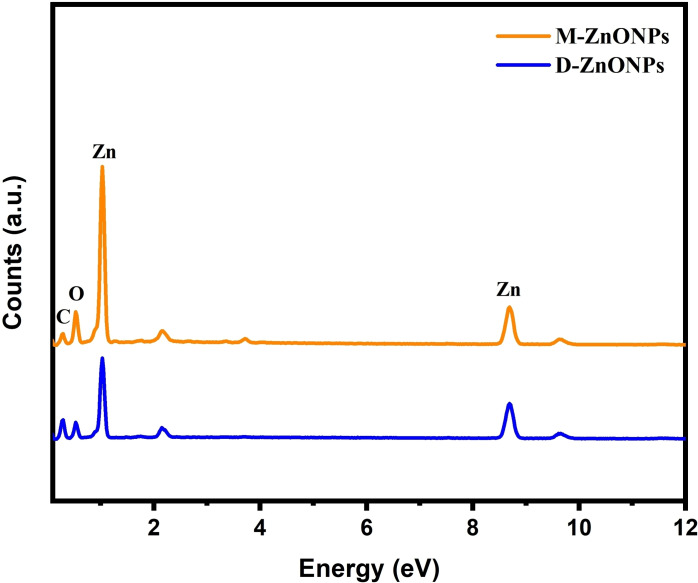
EDX spectra of ZnONPs synthesized using *M. micrantha & D. indica* leaf extract.

To verify the existence of the components and their relative composition in the produced nanomaterials, energy dispersive X‐ray spectroscopy (EDX) analysis was performed. Thus, this research also performed an EDX examination. Figure 6 displays the EDX spectra of D‐ZnONPs and M‐ZnONPs. Both of the synthesized ZnONPs include zinc (Zn) and oxygen (O), as confirmed by their spectrum. It was determined that D‐ZnONPs contained 37.51 and 48.33 % atomic percentages of zinc and oxygen, respectively. On the other hand, M‐ZnONPs showed values of 38.43 % and 46.53 %. Furthermore, phytochemicals capped on the surface of ZnONPs may have been the source of the carbon (C) signal observed in both spectrums. These results are mostly consistent with other research,[^37^, ^39^, ^60^] with minor variances arising from changes in plant chemical compositions.

### Applications of Synthesized ZnONPs

#### Photocatalytic Activity

**Figure 7:**
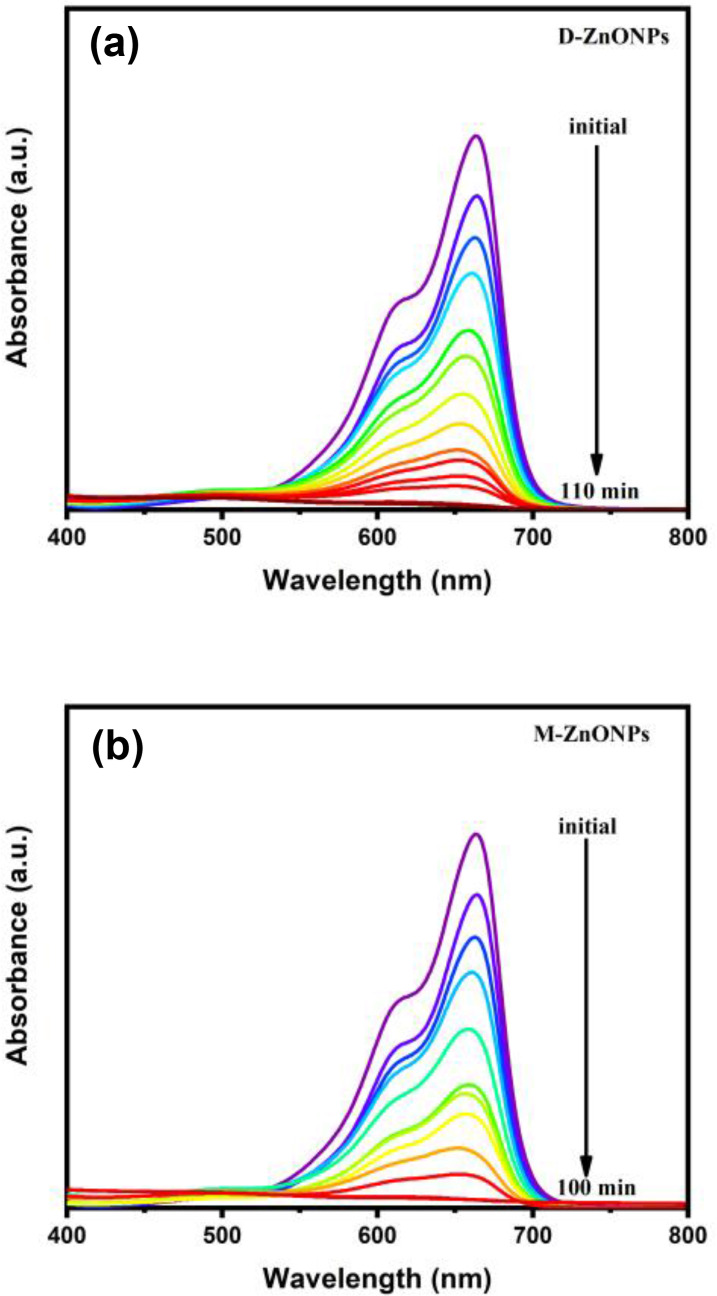
Time‐dependence UV‐Vis spectra for the photocatalytic degradation of MB dye under solar irradiation using ZnONPs synthesized using (a) *D. indica* and (b) *M. micrantha* leaf extract.

ZnONPs are are highly valuable as photocatalysts due to their efficiency in generating reactive oxygen species, broad applicability in environmental remediation, antibacterial properties, and potential for renewable energy applications. Their cost‐effectiveness, stability, and versatility further enhance their importance in various industrial and environmental applications.[^55^, ^56^, ^61^] More presisly, ZnONPs are widely recognized for their potential use in the organic dye degradation process. Thus, methylene blue (MB) dye degradation behavior as a function of solar irradiation was analyzed in this study to examine the photocatalytic activity of ZnONPs synthesized using *D. indica* and *M. micrantha* leaf extracts, and the degradation process was tracked using UV‐Vis spectroscopy. Figure 7 shows the time‐dependent UV‐Vis absorption spectra for the photocatalytic degradation of MB dye utilizing D‐ZnONPs (Figure 7a) and M‐ZnONPs (Figure 7b) under solar irradiation. Figure 7 shows that the distinctive absorption band of MB, which was observed at 664 nm, steadily lost strength and eventually vanished. It takes 110 min for D‐ZnONPs (Figure 7a) and 100 min for M‐ZnONPs (Figure 7b). This outcome is in good agreement with the ZnONPs that have been reported.[^32^, ^42^] According to electron microscopy study, M‐ZnONPs has a significantly smaller particle size, which accounts for its comparatively quick processing time for the degradation of MB dye. Some control experiments were also carried out in the dark, in room light, at 45 °C, and without ZnONPs under solar irradiation for a better understanding of the photocatalytic process. The time‐dependent UV‐Vis absorption spectra are provided in supplemental data (Figure S1–4). The results of control studies indicate that in the absence of photocatalyst, MB dye does not undergo considerable degradation (Figure S1) even under solar irradiation. The observed drop in MB peak intensity can be attributed to MB′s adsorption onto ZnONP surfaces. Additionally, control experiments showed that MB dye did not considerably degrade at 45 °C, in the dark, or even under room light exposure (Figure S2–4). Under such control settings, the proportion of MB dye degradation ranged from 8 to 20 %. The overall result from control tests may contribute to the presumption that photocatalysis is accelerated primarily by UV light from the solar irradiation.

**Figure 8:**
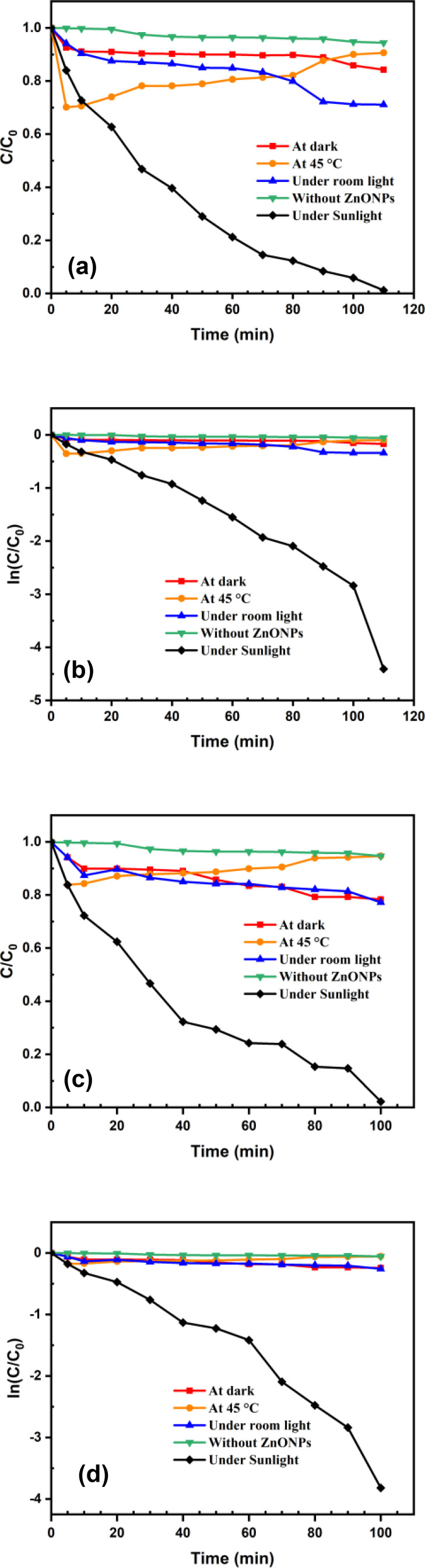
Photocatalytic degradation kinetics of MB dye at various condition using ZnONPs as a photocatalyst: (a) C/C_0_ vs time and (b) ln(C/C_0_) vs time plots for D‐ZnONPs; (c) C/C_0_ vs time and (d) ln(C/C_0_) vs time plots for M‐ZnONPs

The pseudo‐first‐order equation was fitted using experimentally acquired standards to provide the kinetic rate equations of C/C_0_ vs time (min) and lnC/C_0_ versus time (min) as seen in Figure 8. The photodegradation of MB followed pseudo‐first‐order, according to the linearity in the degradation pattern. Seemingly, from the pseudo‐first‐order, apparent rate constants (K1app) of D‐ZnONPs and M‐ZnONPs for the photocatalysis of MB were determined to be 0.0339 min^−1^ (R^2^=0.8969) and 0.0352 min^−1^ (R^2^=0.9509), respectively. In comparison with the reported works, relatively good rate stability results show that both of the ZnONPs are effectively processing dye degradation. Because of their large surface area, ZnONPs have a high photocatalytic activity because they maximize the surface charge interaction with MB dye.[^32^, ^42^, ^44^] Additionally, it appears that M‐ZnONP′s rate constant value is somewhat greater than D‐ZnONP′s. This might be because M‐ZnONP′s particle size is smaller than D‐ZnONP′s, which results in a higher active surface area.

Many research groups have reported on the well‐known process of ZnONPs and organic dyes′ photocatalysis.[^6^, ^40^, ^42^, ^44^] Accordingly, we also explain the mechanism as follows: when ZnONPs are exposed to sunlight, a photo‐generated electron (e^−^) and a hole (h^+^) are created. The excited electrons (e^−^) in the conduction band and the remaining holes (h^+^) in the valence band are the primary active species in the photocatalytic process. The photo‐generated electron (e^−^) subsequently combines with an oxygen molecule to produce a superoxide‐free radical. Extremely unstable hydroxyl radicals are created when the hole (h^+^) interacts with water and hydroxyl ions. The generated reactive oxygen species (ROS), particularly hydroxyl radicals (⋅OH) and superoxide anions (O_2_⋅^−^), are highly reactive and can oxidize a wide range of organic pollutants. These ROS can break down complex organic molecules into simpler, less harmful compounds. The MB was degraded, discolored, and transformed into CO_2_, H_2_O, and other molecules by these free radicals.

#### Antibacterial Activity

**Figure 9:**
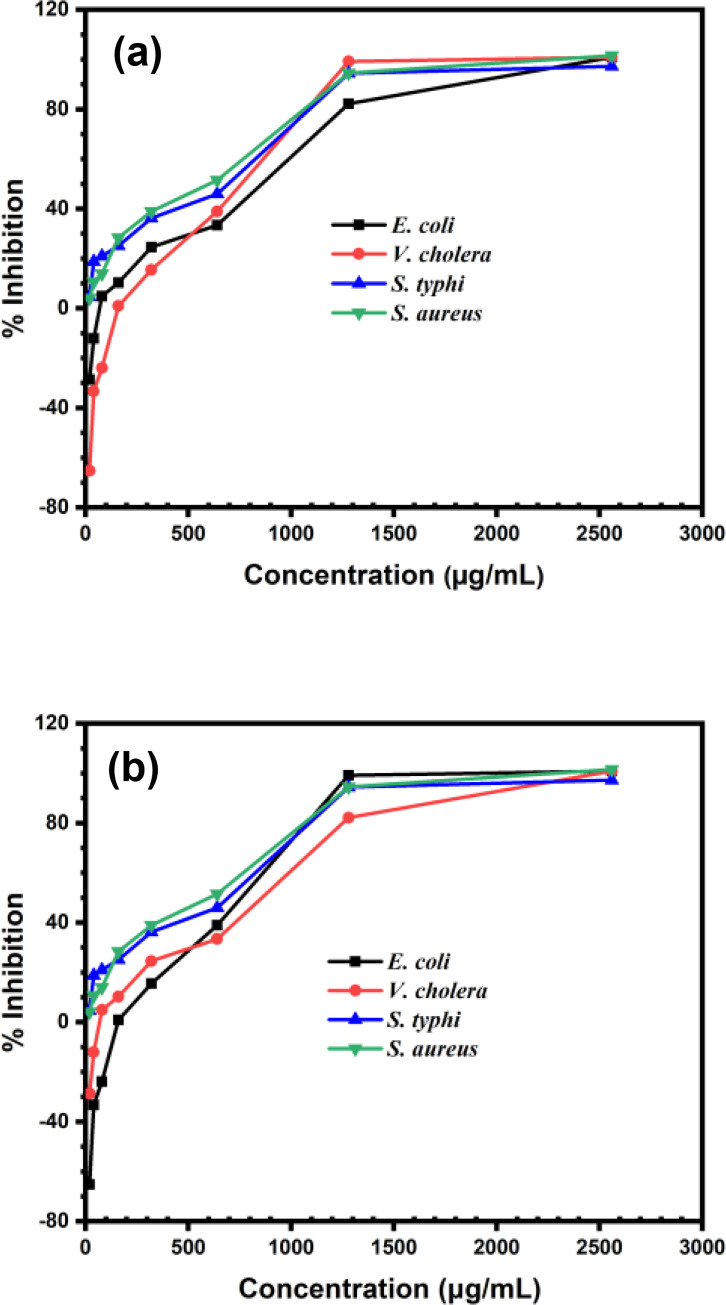
Inhibition percentage of bacterial strains obtained from Resazurin‐based MIC assay by using synthesized (a) D‐ZnONPs and (b) M‐ZnONPs.

Nanoparticles, including ZnONPs, have garnered significant attention as antibacterial agents due to their unique properties and mechanisms of action.[^62^, ^63^, ^64^] ZnONPs are effective against a wide range of bacteria, including both Gram‐positive and Gram‐negative strains. Their multifaceted mechanisms make it difficult for bacteria to develop resistance. ZnONPs can enhance the efficacy of conventional antibiotics when used in combination, allowing for lower doses of antibiotics and reducing potential side effects and resistance development. ZnONPs can be incorporated into various materials and products, such as medical devices, coatings, textiles, and wound dressings, providing long‐lasting antibacterial properties. Moreover, it can be used to disinfect water and surfaces, reducing the spread of bacterial pathogens in the environment and contributing to public health and safety.[^65^, ^66^] The potential of the synthesized ZnONPs as antibacterial agents was assessed by measuring MIC and performing disk diffusion assay. To determine the minimum inhibitory concentration (MIC) values of synthesized ZnONPs, a resazurin assay utilizing a microtiter‐plate was performed against four bacterial strains, namely, *Salmonella typhi* ATCC‐1408 (*S. Typi*), *Vibrio cholerae* ATCC‐51394 (*V. cholerae*), *Staphylococcus aureus* ATCC‐6538 (*S. aureus*), and *Escherichia coli* ATCC‐8739 (*E. coli*). For this assay, 25 μL of each ZnONPs of different concentrations of 2560, 1280, 640, 320, 160, 80, 40 and 20 μg/mL was taken in an individual well of a microtiter plate containing nutrient broth (215 μL), bacterial strains (10 μL), and resazurin (10 μL). Antibiotic (Streptomycin) was used as standard. The optical density (OD) was used to calculate the percentage of inhibitions, and the results are presented in Figure 9. Figure 9 illustrates that for every bacterial strain, inhibition over 80 % was achieved at a concentration of 1280 μg/mL for D‐ZnONPs and M‐ZnONPs, whereas, 50 % of inhibition is considered as the optimum. Therefore, it can be inferred that the MIC for both D‐ZnONPs and M‐ZnONPs is 1280 μg/mL. The obtained MIC values are taken into account to perform the disk diffusion assay.

**Figure 10:**
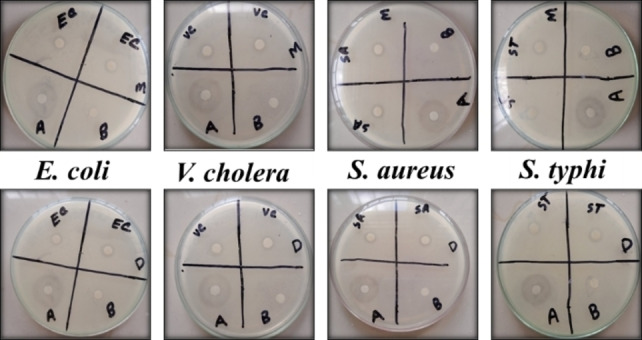
Representation of zone of inhibition (ZOI) of the disk diffusion assay of M‐ZnONPs (upper) and D‐ZnONPs (lower) against bacterial strains.

**Figure 11:**
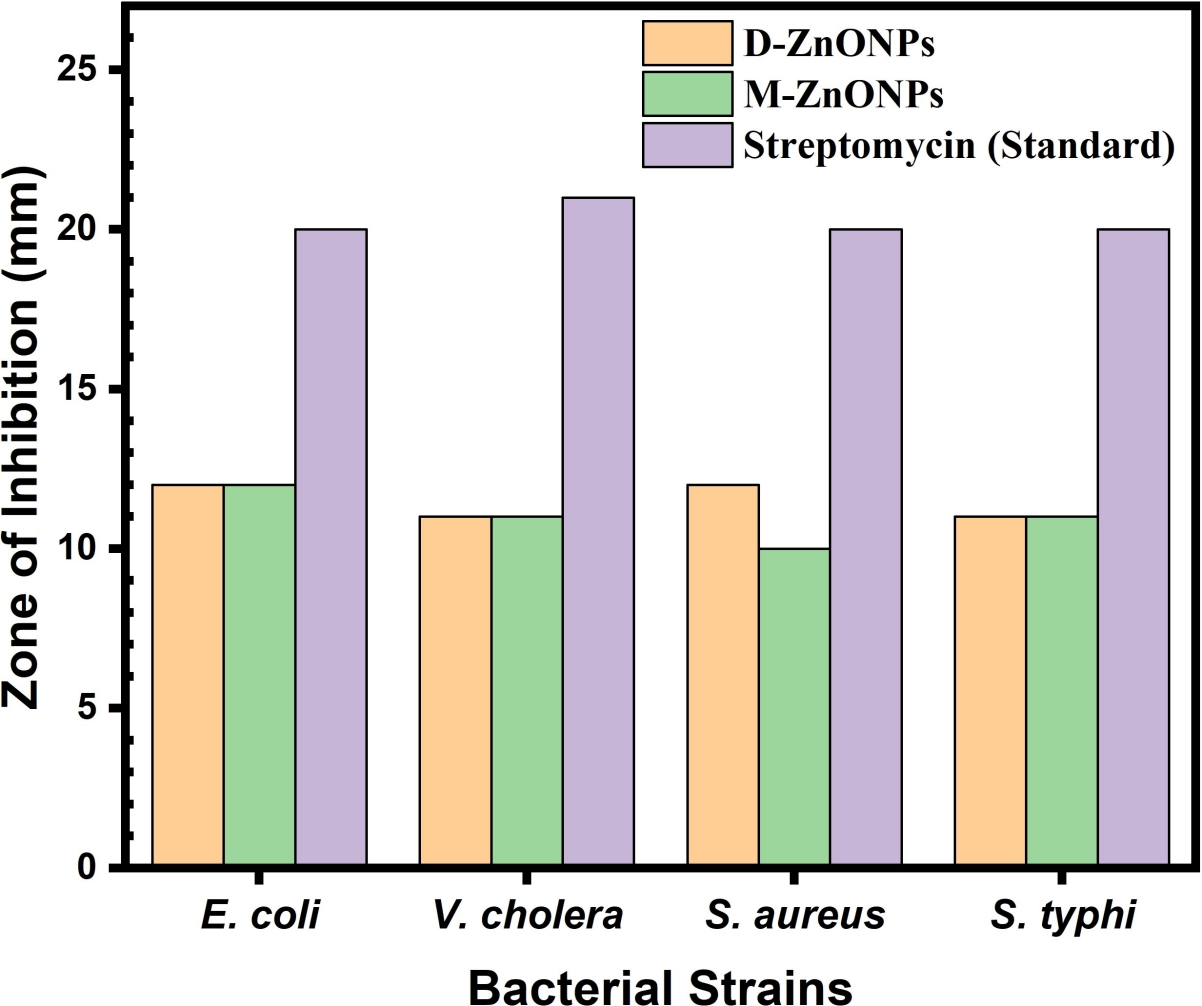
Zone of Inhibition (ZOI) value of bacterial strains obtained from disk‐diffusion assay by using synthesize D‐ZnONPs and M‐ZnONPs.

The disk diffusion assay was run against each of the four bacterial strains using streptomycin as the reference antibiotic, based on the minimum inhibitory concentrations (MIC) found from the resazurin assay. The results were expressed as a zone of inhibition (ZOI) on a mm scale. The depiction of the M‐ZnONPs (upper) and D‐ZnONPs (lower) disk diffusion experiment against bacterial strains is shown in Figure 10, where the following symbols are used: A=antibiotic; B=blank; EC=*E. coli*; VC=*V. cholera*; SA=*S. aureus*; ST=*S. typhi*; D=D‐ZnONPs; and M=M‐ZnONPs. Both D‐ZnONPs and M‐ZnONPs exhibit considerable ZOI, as can be seen in Figure 10, and Figure 11 plots the measured ZOI. M‐ZnONPs and D‐ZnONPs both had almost identical ZOI and were nearly 55–60 % of standard. This outcome is in good agreement with the ZnONPs that have been reported.[^31^, ^42^] The results of the overall investigation of antibacterial activity showed that green synthesized ZnONPs, aided by *D. indica* and *M. micrantha* leaf extracts are relatively cost‐effective, and their incorporation into various products can be economically viable, especially considering their long‐lasting antibacterial effects and had a substantial ability to prevent the development of bacteria and may find use in environmental remediation, promotion of wound healing, cosmetics and ointments.[^6^, ^61^, ^64^]

The disruptive effect of ZnONPs on the cell, which results in increased production of reactive oxygen species (ROS) and several pathways that ultimately lead to growth arrest and microbial cell death, may be the cause of the obstruction to microbial metabolism and growth. Nonetheless, there was significant variation in the level of antibacterial action against various microorganisms. Although the exact process by which ZnONPs affect microorganisms remains unclear, past research suggests that NPs modify the cell wall, increase the porosity of the cell membrane, and ultimately lead to cell death.[^29^, ^31^, ^37^, ^38^, ^41^] Briefly, under light and even in the dark, ZnONPs could produce ROS such as hydrogen peroxide (H_2_O_2_), hydroxyl radicals (⋅OH), and superoxide anions (O_2_⋅^−^). These ROS induce oxidative stress, which ruins lipids, proteins, and DNA in cells and ultimately results in cell death. ZnONPs release Zn^2+^ ions into the culture fluid or adsorb to the cell wall. ZnONPs affect DNA, proteins, and mitochondria in addition to entering the cell by a number of other routes. ZnONPs can harm bacterial cell membranes through physical interactions. This interaction can result from increased membrane permeability, internal cell leakage, and finally cell lysis. Because of their tiny size, the nanoparticles may efficiently penetrate and damage the membrane structure. Moreover, ZnONPs in aquatic settings can liberate Zn^2+^ ions. These ions can enter bacterial cells and disrupt several biological functions. By attaching to the active sites of enzymes or interacting with other essential proteins, zinc ions can impair their functionality and cause metabolic problems and cell death. Bacterial proteins, especially those on the cell surface, can be bound by ZnONPs. Essential protein activity may be inhibited by this interaction, upsetting cellular functions and ultimately resulting in cell death. Additionally, ZnONPs can cause protein aggregation, which may be harmful to the bacterial cell. In addition, the expression of bacterial genes related to metabolism, pathogenicity, and stress response can change when exposed to ZnONPs. As a result, the bacteria may be less able to defend themselves against other antibacterial substances or immunological reactions. ZnONPs′ physical characteristics, including size, shape, and surface charge, are essential to their antibacterial action. The bacterial cell wall and membrane may sustain mechanical damage from sharp edges and large surface areas. Together, these steps may increase ZnONPs′ overall antibacterial activity. One major benefit of ZnONPs over standard antibiotics is their multidimensional approach, which makes it harder for bacteria to acquire resistance to them.[^65^, ^66^]

## Conclusions

Utilizing *D. indica* and *M. micrantha* leaf extract, two ZnONPs, denoted as D‐ZnONPs and M‐ZnONPs, have been effectively synthesized in the current work employing a green methodology. Using different characterizing tools, the produced ZnONPs were evaluated in terms of their morphological, structural, and optical characteristics. The crystalline wurtzite structure of ZnONPs was disclosed during the process of ZnONP characterization. ZnONPs were found to have both nearly spherical and irregular shape, and the spherical one have the particle sizes of 26 nm for M‐ZnONPs and 30 nm for D‐ZnONPs. ZnONPs′ photocatalytic activity was examined in relation to MB dye. According to the observed result, photocatalysis using both of the ZnONPs follow pseudo‐first‐order kinetics and degrade MB within 100 min at 0.0352 min^−1^ of rate constant by M‐ZnONPs, whereas D‐ZnONPs degrade MB within 110 min at 0.0339 min^−1^ of rate constant. Furthermore, it has been shown that both ZnONPs have noteworthy efficacy against the following bacterial strains: *Salmonella typhi* (ATCC‐1408), *Vibrio cholerae* (ATCC‐51394), *Staphylococcus aureus* (ATCC‐6538), and *Escherichia coli* (ATCC‐8739). Because of its excellent antibacterial activity, remarkable photocatalytic ability and excellent stability, current studies encourage the suggestion of using green strategy‐based ZnONPs in various industrial and environmental applications, especially in wound healing, wastewater treatment and cosmetics industries. It also promotes the use of green synthesis methods in the production of other nanomaterials.

## Experimental Section

**Preparation of plant extract**. In Khulna, Bangladesh, on the campus of Khulna University, fresh leaves of *M. micrantha* and *D. indica* were harvested. After being meticulously chopped into tiny bits, the leaves were cleaned in deionized distilled water (DDW) and left to air dry in the lab. The mentioned procedures were somewhat modified to prepare the leaf extracts.[^20^, ^67^] A separate round‐bottom flask holding 500 mL of DDW was filled with 30 g of dried *M. micrantha* and 10 g of dried *D. indica* leaves. The mixture was then brought to a boil for 60 min. After boiling, the mixes were allowed to cool to room temperature. The filter paper was used to filter the extracts, and centrifugation was performed at 10,000 rpm. Lastly, the extracts were stored for later use at 4 °C in a refrigerator.

**Materials**. Zinc acetate dihydrate (Zn(CH_3_COO)_2_ ⋅ 2H_2_O) with a purity of >99.0 % was purchased from Sigma‐Aldrich (Germany). The source of methylene blue was Kanto Chemical Co. Inc. (Tokyo, Japan). Sodium hydroxide (NaOH) pallets, ethanol, hydrochloric acid (HCl), nitric acid (HNO_3_), and acetone were purchased from Merck‐India. The necessary solutions for the synthesis of materials were made in deionized water, and all of the reagents were employed without additional purification. The Biotechnology and Genetic Engineering Discipline at Khulna University in Bangladesh provided the nutrient broth, nutrient agar medium, and bacterial stain *E. coli*, *S. aureus*, *V. cholerae*, *S. typhi*, and antibiotic streptomycin.

**Synthesis of ZnONPs**. Previously published procedures were slightly modified to synthesize ZnONPs.[^31^, ^39^, ^42^] In order to synthesize ZnONPs, 50 mL of leaf extract was combined with 2.0 g of zinc acetate dihydrate, and the mixture was stirred for 2 h at 60 °C using a magnetic stirrer. After the reaction was finished, the mixture was allowed to cool at ambient temperature and centrifuged at 10,000 rpm. Subsequently, the collected product underwent a 2 h calcination at 500 °C, yielding a gray powder substance. This powder underwent multiple DDW washes before being oven‐dried at 90 °C. Two annealed products were saved at 4 °C for later use; they were designated as D‐ZnONPs and M‐ZnONPs when using *D. indica* leaves extract *M. micrantha* leaf extract, respectively.

**Characterization of synthesized ZnONPs**. Several characterization techniques have been used for green synthesized ZnONPs to examine their physiochemical characteristics. These techniques include ultraviolet (UV) and Fourier‐transform infrared (FTIR) spectroscopy, X‐ray diffraction (XRD), and energy‐dispersive X‐ray (EDX) analysis, scanning electron microscopy (SEM), and transmission electron microscopy (TEM). A UV‐1900i UV‐Vis spectrophotometer from SHIMADZU (Tokyo, Japan) was used for the UV‐Vis spectroscopic study. Within the range of 200–800 nm, absorption spectra were collected at a resolution of 1 nm. At a temperature of 25 °C, FTIR spectra were captured on an IRSpirit Fourier transform infrared spectrophotometer by SHIMADZU (Tokyo, Japan) using a KBr pellet at a scan rate of around 4 cm^−1^ s^−1^. On a Hitachi (Tokyo, Japan) SU‐8000 microscope, SEM measurements and EDX analysis were carried out at accelerating voltages of 10 and 15 kV. A TEM‐2100F field emission electron microscope (JEOL, Tokyo, Japan) was used for the TEM measurements. A Cu−Kα radiation‐based SmartLab spectrometer from Rigaku (Tokyo, Japan) was used for the XRD study.

**Photocatalytic activity**. Previously published procedure was modified to evaluate the photocatalytic activity of synthesized ZnONPs.[^32^, ^42^] In order to assess the photocatalytic activity of the green synthesized ZnONPs, a 5 mg/mL methylene blue dye solution was prepared in 100 mL of DDW, and 50 mg of ZnONPs catalyst was added to the dye solution. The mixture was placed at dark for 30 min to achieve the adsorption–desorption equilibrium. The solution was then exposed to sunlight and collect 4 mL of the reaction mixture at 10 min interval. A UV‐Vis ible spectrophotometer was used to monitor the dye degradation while the catalyst was separated from the mixture using centrifugation at 10,000 rpm for 5 min. The following equation was used to determine the percentage of the dye that had degraded.

(4) $$\%\;of\;Degradation=\;\frac{C_{0}-C_{t}}{C_{0}}\;\times 100$$

Where $C_{0}$ is the initial concentration and $C_{t}\;$ is the concentration after a time interval.

**Antibacterial activity**. It was looked into if ZnONPs generated from plant leaves might stop the growth of harmful microorganisms. With the help of the Resazurin‐based Minimum Inhibitory Concentration (MIC) study and the agar well diffusion method, the following strains–*Salmonella typhi ATCC‐1408 (S. Typi), Vibrio cholerae ATCC‐51394 (V. cholerae), Staphylococcus aureus ATCC‐6538 (S. aureus), and Escherichia coli ATCC‐8739 (E. coli)*–were used to evaluate the antibacterial activity.

**Resazurin**‐**based Minimum Inhibitory Concentration (MIC) analysis**. According to the procedures reported,[^37^, ^38^] using a Resazurin‐based MIC study, the MIC values were ascertained During the experiment, 215 μL of nutritient broth and 25 μL of the obtained ZnONPs with concentrations of 2560, 1280, 640, 320, 160, 80, 40, and 20 μg/mL were applied to each well of the microtiter plate. The same proportion of streptomycin, the common antibiotic, was added. Next, 10 μL of bacterial strains were added to each well of the microtiter plate, omitting the blank row. 10 μL of resazurin was added to each well and incubated for 30 minutes after the initial 24 h incubation at 37 °C. MIC was calculated in relation to the microplate reader‘s optical density (OD) reading.

The percentage of inhibition of the tested ZnONPs were calculated according to the following equation:

(5) $$\%\;of\;Inhibition=\;\frac{Absorbance\;of\;blank-Absorbance\;of\;sample}{Absorbance\;of\;Blank}\times 100$$

**Disk Diffusion Assay**. The disk diffusion assay was carried out according to the procedures reported.[^42^, ^43^] In a typical procedure, the Whatman No. 1 filter paper was punched with a punching machine to prepare the sample disk, which has a diameter of 6 mm. After being placed within the screw‐capped tube, the disks were autoclaved for 15 min at 121 °C and 15 lb/inch^2^ of pressure. Following this, 10 μL of a 5 mg/mL ZnONPs dispersion was added to the disks. Subsequently, the disks were kept in an aseptic state for few hours to ensure the solvent was completely removed. The disk paper was prepared for the test of its antibacterial activity and was in the vial. Similarly, streptomycin was used to prepare the standard disk as the positive control, while solvent alone was used to prepare the blank disk as the negative control. Using sterile forceps to guarantee contact with the media, the sample disk, standard disk, and blank disk were carefully put on solidified agar plates seeded with the pathogens. In order to give the nanoparticles absorbed into the disk ample time to disperse into the medium, the plates were then left in the laminar for 10 min. The petri dishes were then incubated for 24 h at 37 °C, and the zone of inhibition (ZOI) was determined on a millimeter scale.

## Supporting Information

The authors have a word file for the Supporting Information.

## Conflict of Interests

The authors declare no conflict of interest.

1

## Supporting information

As a service to our authors and readers, this journal provides supporting information supplied by the authors. Such materials are peer reviewed and may be re‐organized for online delivery, but are not copy‐edited or typeset. Technical support issues arising from supporting information (other than missing files) should be addressed to the authors.

Supporting Information

## Data Availability

The data that support the findings of this study are available from the corresponding author upon reasonable request.
