# Supplementary material for: Green Synthesis of Zinc Oxide Nanoparticles Using Dillenia Indica and Mikania Micrantha Leaf Extracts: Applications in Photocatalysis and Antibacterial Activity
Source: ChemistryOpen. 2024 Oct 2;13(12):e202400102. doi: 10.1002/open.202400102 (PMC11625939; doi:10.1002/open.202400102)
Supplement: Supplementary file 1 — Supporting Information [file OPEN-13-e202400102-s001.pdf]

ChemistryOpen

Supporting Information

Green Synthesis of Zinc Oxide Nanoparticles Using *Dillenia Indica* and *Mikania Micrantha* Leaf Extracts*:* Applications in Photocatalysis and Antibacterial Activity

Protap Kumar Pal, Md. Sarifujjaman, Prianka Saha, S. M. Mahbubur Rahman, Md. Emdadul Islam, Bashir Ahmmad, Kaykobad Md. Rezaul Karim, and Md. Mahiuddin*

Supplementary Information

Green Synthesis of Zinc Oxide Nanoparticles Using *Dillenia Indica* and *Mikania Micrantha* Leaf Extracts: Applications in Photocatalysis and Antibacterial Activity

Protap Kumar Pal[1], Md. Sarifujjaman[1], Prianka Saha[1], S.M. Mahbubur Rahman[2], Md. Emdadul Islam[2], Bashir Ahmmad[3], Kaykobad Md. Rezaul Karim[1], and Md. Mahiuddin[1*]

[1]Chemistry Discipline, Khulna University, Khulna-9208, Bangladesh

[2]Biotechnology and Genetic Engineering Discipline, Khulna University, Khulna-9208, Bangladesh

[3]Graduate School of Science and Engineering, Yamagata University, 4-3-16 Jonan, Yonezawa 992-8510, Japan

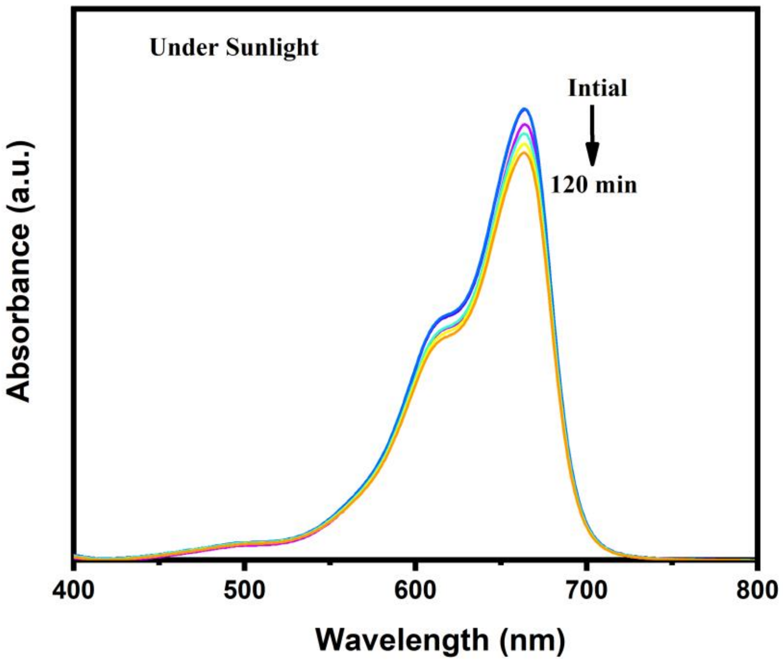

Figure S1. Time-dependence UV-Vis spectra for the photocatalysis of MB dye under solar irradiation in absence of ZnONPs synthesized using *D. indica* and *M. micrantha* leaf extract.

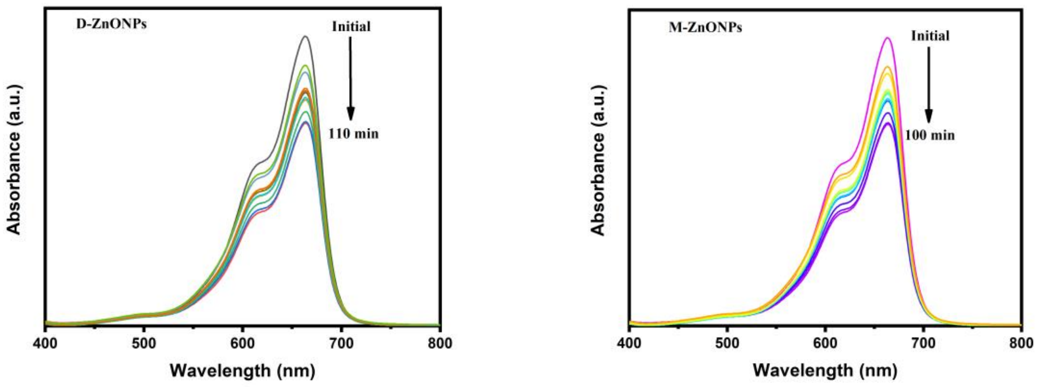

Figure S2. Time-dependence UV-Vis spectra for the photocatalysis of MB dye at 45 °C in presence of ZnONPs synthesized using *D. indica* and *M. micrantha* leaf extract.

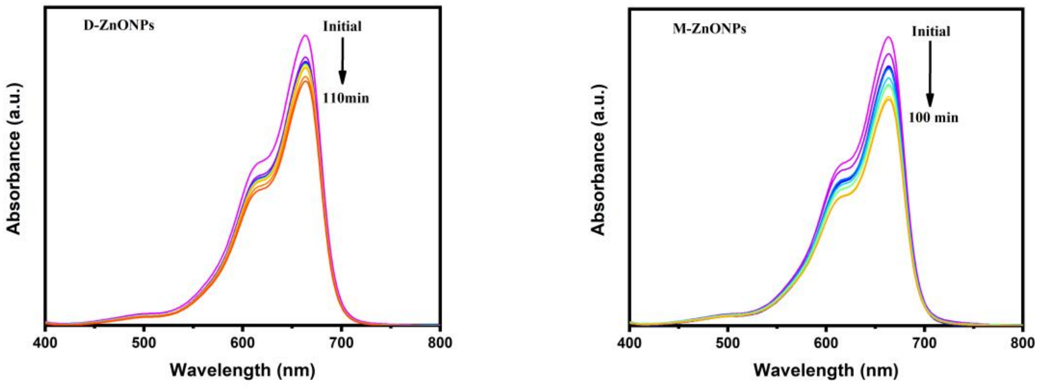

Figure S3. Time-dependence UV-Vis spectra for the photocatalysis of MB dye in the dark in presence of ZnONPs synthesized using *D. indica* and *M. micrantha* leaf extract.

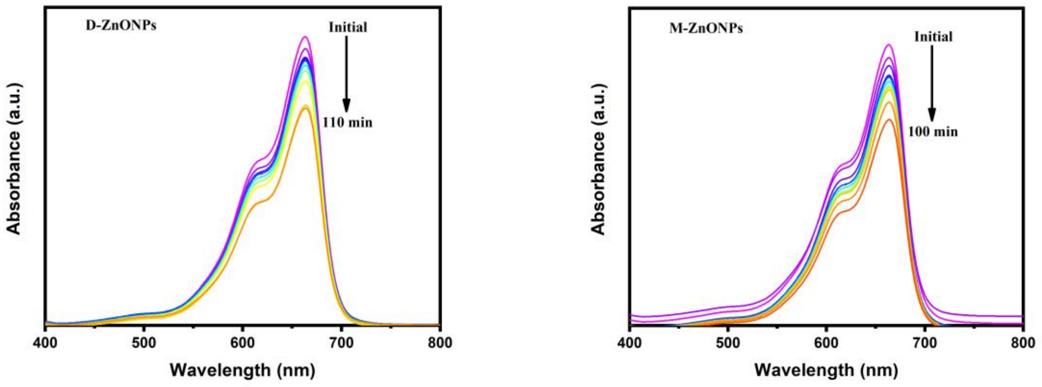

Figure S4. Time-dependence UV-Vis spectra for the photocatalysis of MB dye under room light in presence of ZnONPs synthesized using *D. indica* and *M. micrantha* leaf extract.
